# Effectiveness of Safe Patient Handling Equipment and Techniques: A Review of Biomechanical Studies

**DOI:** 10.1177/00187208231211842

**Published:** 2023-11-10

**Authors:** Mike Fray, Kermit G. Davis

**Affiliations:** 1 152592 Loughborough University, Loughborough, Leicestershire, UK; 2 2514 University of Cincinnati, Cincinnati, OH, USA

**Keywords:** repositioning, lateral transfers, patient handling, spine loading, biomechanics

## Abstract

**Objective:**

This review aimed to evaluate all studies that have evaluated the biomechanical effects when using assistive devices.

**Introduction:**

The physical demands of patient handling activities are well known. One safety strategy for the reduction of the physical risks is use of assistive devices.

**Method:**

The search process identified articles published in English-speaking journals through Google Scholar, Medline, and ISI Web of Science. The included 56 studies contained a biomechanical assessment of a patient handling activity with assistive devices.

**Results:**

The biomechanical effects included four groups: changes in body posture (spinal, other joints), subjective assessment (force, effort, discomfort), measured force (hand force, ground reaction force, spine force, joint torque), and physiological measures. The evidence showed caregivers benefited from using lift hoists, air-assisted devices, and to a lesser extent friction reducing devices for lateral transfers and repositioning, while floor and ceiling lifts were most effective for patient transfers. Some gaps were noted in the evidence and other handling tasks such as sit-to-stand, turning patient in bed, limb lifting, and repositioning and some more high hazard activities like supporting people with limited balance and those that fall need to be investigated with respect to biomechanical outcomes.

**Conclusion:**

There is a growing level of biomechanical evidence to support the use of assistive devices for many patient-handling tasks, but the benefits of equipment use in some transfers remain uninvestigated.

**Practical Application:**

Evidence indicates the best way to lift patients safely is with floor or ceiling lifts, and air-assisted devices for lateral and repositioning tasks.

## Introduction

The physical demands required to assist patient movement are both a common nursing activity and have long been recognized (Garg & Owen, 1992; Jäger et al., 2013; Marras et al., 1999). The relationship between the frequent exposure to high load patient handling tasks is a known contributory factor to the musculoskeletal problems of care workers (Davis & Kotowski, 2015). The developing knowledge and practices consider the use of assistive devices as a recognized control measure to reduce the burden on the care worker (Anderson et al., 2014). The direct reduction of force requirements or the replacement of human effort with mechanical means has received strong support and wide implementation. The range of devices commonly seen in care situations can include: the replacement of full weightlifting with a powered lifter (Alamgir et al., 2009; Dutta et al., 2012), the reduction of forces for horizontal transfers with friction reduction devices (Waters, 2011), patient turning (Budarick et al., 2020), or the use of a powered bed to assist repositioning (Zhou & Wiggermann, 2021).

Several literature reviews have cumulated the body of evidence surrounding both the deleterious effects of patient handling and the possible benefits of a range of available interventions (Al Johani & Pascua, 2019; Mayeda-Letourneau, 2014; Nelson & Baptiste, 2006; Teeple et al., 2017). It should be noted that the reported evidence for the use of safety interventions in patient handling risk reduction is in a development phase and early reviews found only small numbers of high-quality papers for inclusion (Bos et al., 2006; Dawson et al., 2007; Martimo et al., 2008), a more inclusive review reported a much higher number of studies (Hignett et al., 2003) but acknowledges a different and less stringent inclusion criteria. The growth of evidence over a decade is reported in better quality of data and analysis in more recent studies (Anderson et al., 2013; Teeple et al., 2017). Even with the increased knowledge that assistive devices can significantly reduce the load on caregivers, some studies still show that the use of devices is not universal (Koppelaar et al., 2011).

Previous reviews of safe patient handling have focused on the effectiveness of programs and lift equipment in reducing musculoskeletal disorders (MSDs) in healthcare system (Al Johani & Pascua, 2019, Hignett et al., 2003; Mayeda-Letourneau, 2014; Nelson & Baptiste, 2006; Teeple et al., 2017). To understand the effectiveness of safe patient handling equipment, a comprehensive assessment of the studies that have investigated the impact on biomechanical responses within the body during safe patient handling tasks is needed. The aim of the review is to collectively report all the biomechanical effects that have been assessed when using assistive devices and lifting training (e.g., proper lifting technique) across all the range of regularly performed patient transfers and movement activities (Crowshaw and Fray, 2018; Smith et al., 2011). Biomechanical assessments can provide a better understanding of the results as compared to epidemiological studies with respect to safe patient handling equipment being protective of MSDs. The accumulated information could be used to further support the purchase, implementation, and use of assistive devices to better protect care workers from musculoskeletal disorders.

## Method

### Review Approach

The research team searched for published articles (prior to March 2023) that investigated biomechanical outcomes (either subjective or objective) when assessing safe patient handling devices and techniques (e.g., proper lifting). The review was completed using the PRISMA 2020 checklist to ensure quality and reproducibility (Page et al., 2021). Epidemiological studies that did not include biomechanical outcomes were eliminated from consideration, specifically studies that focused on reduced injuries and reported cases were excluded. Others have provided reviews that focused on these outcomes (Anderson et al., 2013; Bos et al., 2006; Dawson et al., 2007; Martimo et al., 2008; Teeple et al., 2017).

### Article Inclusion Criteria

While the following will provide the details of inclusion, the *inclusion criteria* was any biomechanical study that evaluated patient handling equipment and proper lifting techniques. A search for all articles published in English-speaking journals was undertaken through Google Scholar, Medline, and ISI Web of Science. For each of the search engines, the search strategy was to search for the same string of keywords with no filters. The search words included “safe patient handling,” which yielded 13,444 articles. All duplicate articles among the search results were removed. The remaining articles were further screened (by KD) where the title and abstract were assessed for inclusion of biomechanical outcomes (generally) as well as assessment of lifting equipment and proper manual lifting training technique. All articles that meet basic biomechanical assessment were downloaded for additional evaluation of the quality of the study and inclusion of at least one of the specific biomechanical outcomes. Reviews and epidemiological studies investigating the use of lift equipment and safe patient handling programs only were not included in the current review.

As a result of this initial review, included article count was reduced to 102 relevant articles based on the criteria that some type of biomechanical assessment, either objective or subjective, was utilized and described in the abstract. One of the following outcomes had to be included as an outcome variable to be included in the review: spine posture (flexion, lateral flexion, twist in degrees), whole body motion based on motion capture (joint angles in degrees), specific body movement by other joint position method, physiological measures (fatigue with changes of median frequency of muscle activation, oxygen consumption in ml/min, energy expenditure in Kcal), subjective forces (rating of perceived exertion in Likert scale), discomfort (Likert scale, usually 1–10), ground reaction force (three-dimensional force in N), hand or applied force (force in N), muscle activity (normalized in %MVC), spine loads three dimensional loads in N), and joint torque (moment in Nm). These categories served as the classifications to group the studies for synthesis. Each category included specific biomechanical measures as defined by the individual studies.

The next step was to complete a thorough review of the remaining articles to ensure biomechanical assessment and inclusion (completed by KD and verified independently by MF). Articles were only eliminated if they did not have a biomechanical assessment in one or more of the above categories or did not assess lift equipment/proper lifting training. Finally, the reference lists for all relevant articles were scanned to identify any missing articles, which the above process was undertaken for those identified articles. Starting with a broad search of “safe patient handling” and narrowing with the biomechanical outcomes ensured a broad and comprehensive assessment of the literature for patient handling equipment and proper lifting training.

**Figure 1.**
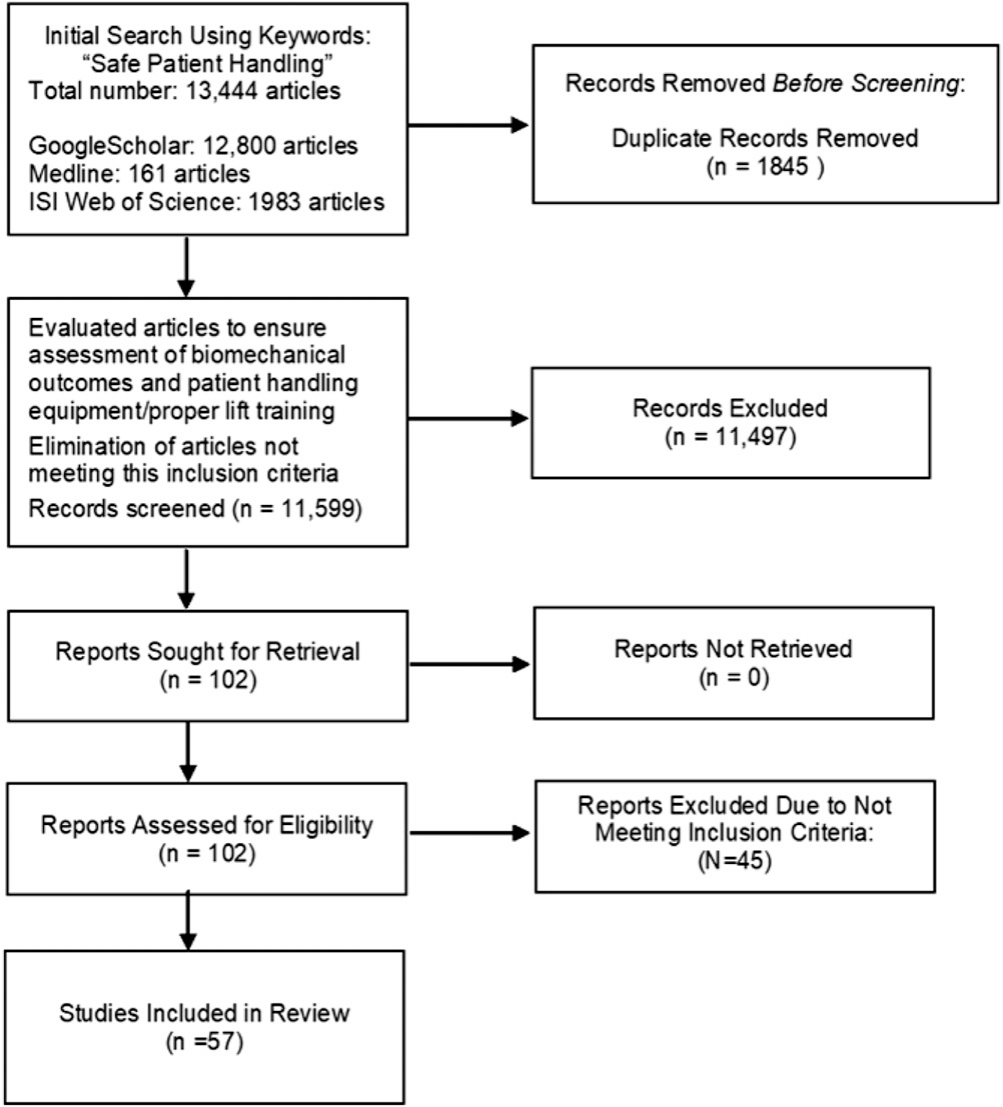
Schematic summary of the article search to identify articles included in the review (Figured adapted from Page and associates, 2021).

In total, 57 articles were included in the review and underwent the quality assessment (completed by KD and verified by MF) (see below for details of quality assessment). The review included all relevant articles with no exclusion due to poor quality, although quality was discussed. Figure 1 provides a schematic of the inclusion process.

### Quality Assessment of Papers

Mixed Methods Appraisal Tool (MMAT), developed by Pluye and Hong (2014), was used to rate the quality of each article. Based on the criteria for qualitative and quantitative random designs, quantitative nonrandom designs, and mixed methods, each article was rated and scored (by KD, and independently verified by MF). The main questions rated were: (1) Are the participants representative of the target population? (2) Are there complete outcome data? (3) Are the confounders accounted for in the design and analysis? (4) Are outcome assessors blinded to the intervention provided? and (5) Did the participants adhere to the assigned intervention? The articles were then quantified based on the score (each yes for the questions associated with the study design) and whether measurements were valid. The measurement evaluation was based on criteria: sufficient numbers of participants (greater than 20), measures were validated in literature—techniques well established with reliability quantitative measures with calibrated equipment and techniques, using realistic patients—assessments using standardized, noncompliant, realistic patients, and reliable equipment—use of sensitive equipment and surveys. These additional criteria assess the quality of measures and whether conditions were realistic to healthcare settings. Each of the criteria was rated as satisfactory/yes (1) or not satisfactory/no (0) with the total scores providing the overall quality assessment. Based on these scores, the articles were classified in the following quality categories: (1) low (score 0–1), (2) low-medium (score 2–3), (3) medium (score 4–5), (4) medium-high (score 6–7), and (5) high (score 8–9).

### Synthesis Methods for Review

In order to evaluate all existing literature for safe patient handling equipment and proper lifting training, all 57 articles were included with none being eliminated. To understand the details of studies included in the review, Table 1 included authors, dates of publication, study population, country where study was collected, study design, and the quality rating of the study was constructed. The study design was particularly important in synergizing the results of the studies as it indicated the outcome measures used and the type of equipment/training. For each of the studies, the team tabulated the results for each biomechanical outcome variable that was measured as a function of the safe patient handling equipment and lifting technique training (see Appendix for the complete results). Based on the results in the tables in the Appendix, a summary table was developed to provide a more global summary of the results so that a more complete assessment of the existing results could be identified (see Table 2).

**Table 1.** Summary of Studies Included in the Biomechanical Review: Author, Date, Study Population, Country, Study Design, and Rating of Study Quality

Author	Date	Study Population	Country	Study Design	Study Quality Rating
Allen, Jackson, Marsden, McLellan, Gore	2002	6 female trained nurses	United Kingdom	*Laboratory study*: Evaluated compression, for transferring devices: no equipment, belts, turntables, slings, and transfer boards	Medium
				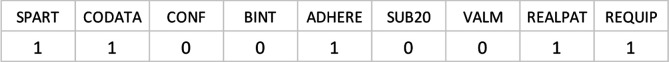	
Al-Qaisi, Tannir, Younan, Kaddoum	2020	16 male nurses	Lebanon	*Laboratory study*: Evaluated muscle activity and perceived exertion when transferring from operating table (tilted and flat) with a draw sheet, a plastic bag, and a slide board	Low-medium
				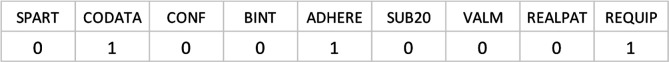	
Bacharach, Miller, von Duvillard	2016	3 clinical nurses from the university who were trained in patient transfer	United States	*Laboratory study*: Evaluated frictional forces for transfer devices (roller tray slide board, nylon tube, and roller board)	Low-medium
				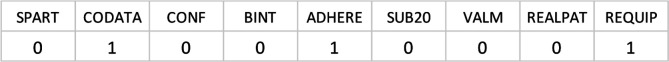	
Bartnik, Rice	2008	29 male and female participants	United States	*Laboratory study*: Evaluated hand forces, compression, and shear forces for three friction reducing sheets (disposable fabric slide sheet and MaxiSlide sheet) versus draw sheet when performing repositioning	Medium
				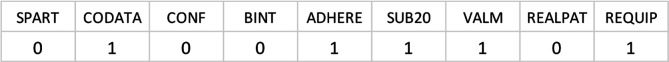	
Blaauw, Greenhalgh, Vegter, Bass, Kulich, Grindle, Cooper, Koontz, Cooper	2020	20 caregivers (15 female and 5 male)	United States	*Laboratory study*: Evaluated muscle activity of erector spinae, latissimus dorsi, sternal portion of the pectoralis major, and anterior deltoid when transferring patients (toilet, bench, and shower chair) with the strong arm and Hoyer Advance	Medium
				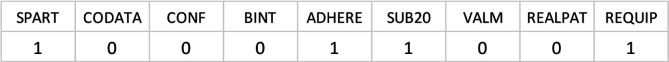	
Budarick, Lad, Fischer	2020	17 female and 8 male caregivers	Canada	*Laboratory study*: Evaluated hand force, shoulder and trunk moment, and spine force when using turn-assist surfaces relative to manual patient turns	High
				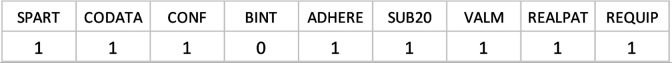	
Cheung, Dai, Cheung, Cho, Chow, Fung, Lam, Lia, Ng, Ngan, Szeto	2020	10 female nursing students	China	*Laboratory study*: Evaluated kinematics, muscle activities, and perceived physical exertion during patient transfers between a bed and a wheelchair, with or without a transfer belt	Low-medium
				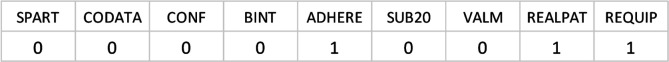	
Daynard, Yassi, Cooper, Tate, Norman, Wells	2001	36-unit assistants	Canada	*Field study*: Assessment of spine force during transfer tasks: Bed-to-wheelchair transfer, bed-to-stretcher transfer, bed boost, chair boost, and bed turn using two size people: 55-kg weight bearing cooperative and 100 kg passive	Medium-high
				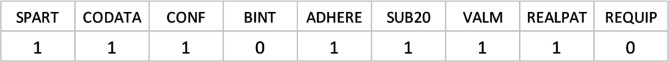	
Drew, Kozey, Moreside	2015	10 females	Canada	*Laboratory study*: Evaluated low back muscle activity, hand force, and perceived effort when using standard sheet, slide sheet, and modified slide sheet.	Low-medium
				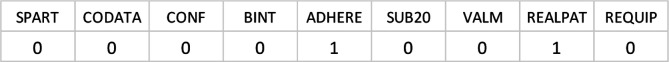	
Dutta, Holliday, Gorski, Baharvandy, Fernie	2011	21 female caregivers	Canada	*Laboratory study*: Evaluated peak external forces and moments generated at the L5/S1 joint when maneuvering loaded floor-based and overhead mounted patient lifting devices for bed to wheelchair and back to a bed	Medium-high
				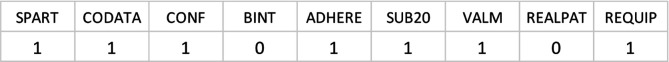	
Dutta, Holliday, Gorski, Baharvandy, Fernie	2012	21 caregivers had at least one year experience in patient lift/transfer activities	Canada	*Laboratory study*: Measured hand forces and external moments at the L5/S1 joint when moving a patient from a bed to a wheelchair and back to a bed using floor and overhead lifts	Medium-high
				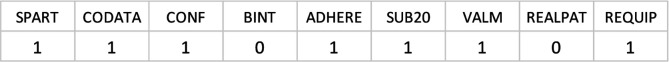	
Elford, Straker, Strauss	2000	22 female nurses	Australia	*Laboratory study*: Measured trunk kinematics while transferring patient from chair using two-person lift with and without slings	Medium-high
				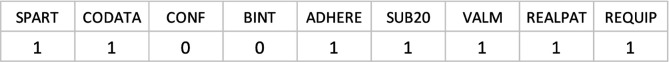	
Gagnon, Sicard, Sirois	1986	6 male physical education students	Canada	*Laboratory study*: Estimated spine loads and mechanical work and energy during patient handling tasks	Low-medium
				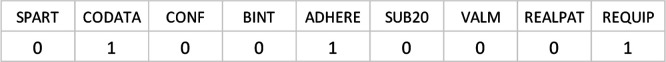	
Garg, Owen	1992	38 nursing aides (36 females and 2 males)	United States	*Field study*: Measured the pain levels, static compression, and perceived stress when using lifting hoist and walking belt in a nursing home	Medium
				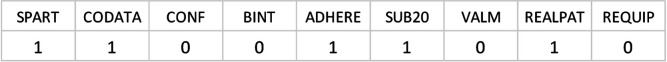	
Garg, Owen	1994	Laboratory study: 6 female nursing students with at least 1-year experienceField study: 57 nursing assistants	United States	*Laboratory study*: Estimated compression forces and measure trunk posture when lifting by two persons, gently rocking and pulling the patient with a gait belt and walking belt by two persons and rocking and pulling the patient with a walking belt and using a sling by one person and three mechanical hoists.*Field study*: Investigated use of ergonomic handling equipment: Hoist, lifting belt on injury rates, perceived exertion and acceptability rates	Medium
				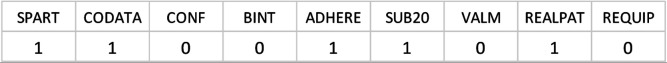	
Garg, Owen, Beller, Banaag	1991a	6 female nursing students	United States	*Laboratory study*: Measured pull forces and estimated spine compression and shear forces when transferring patients to and from the bed with and without lifting assist devices	Medium
				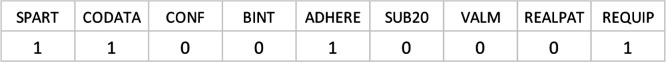	
Garg, Owen, Beller, Banaag	1991b	6 female nursing students	United States	*Laboratory study*: Measured pull forces and estimated spine compression and shear forces when transferring patients between a wheelchair and shower with and without lifting assist devices	Medium
				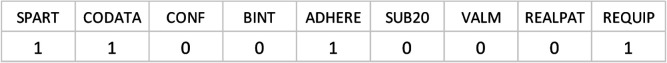	
Grevelding, Bohannon	2001	2 men	United States	*Laboratory study*: Measured push force when using slide devices (none, easy glide, Minislide or both) while pushing a passive patient	Medium
				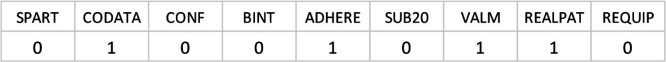	
Hess, Kincl, Mandeville	2007	16 clinicians	United States	*Laboratory study*: Measured trunk kinematics, perceived exertion, and hand force for modified standing pivot, a transfer board technique, and the scoot transfer	Medium
				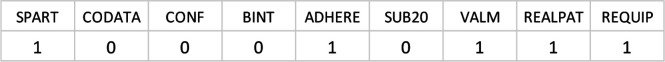	
Hodder, MacKinnon, Ralhan, Keir	2010	12 female untrained individuals and 10 female experienced nurses	Canada	*Laboratory study*: Measured muscle activity and trunk posture during 3 patient transfers comparing trained and untrained individuals in proper body mechanics	Medium
				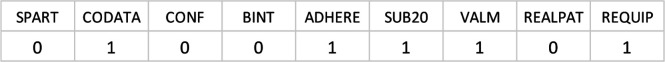	
Howard, Bao, Kim, Silverstein	2013	2 female caregivers	United States	*Laboratory study*: Measured muscle activity during transfer tasks using ceiling lift, scoot transfer, modified scoot transfer, and manual lift	Low-medium
				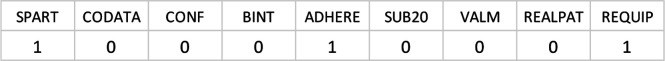	
Hwang, Ari, Matoo, Chen, Kim	2020	20 professional caregivers (18 females and two males)	United States	*Laboratory study*: Measured muscle activity, trunk posture, and trunk moment during turning tasks in two turning directions (toward vs. away relative to caregivers) using five device conditions: Draw sheet, friction-reducing turning sheet, air-assisted transfer device, air-assisted turning device, and no assistive device	Medium-high
				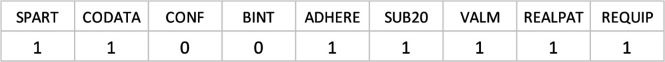	
Hwang, Kuppam, Chodraju, Chen, Kim	2019	20 professional caregivers (18 females and 2 males)	United States	*Laboratory study*: Measured hand force; shoulder and trunk posture; shoulder moment; muscle activity and usability ratings from four devices: a Draw sheet, a repositioning sheet, a slide board, and an air-assisted device during repositioning	Medium-high
				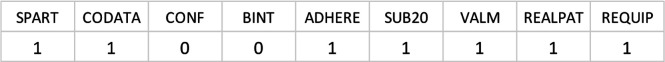	
Iridiastadi, Vani, Yamin	2020	12 nurses who worked at local hospital for at least a year	Indonesia	*Laboratory study*: Measured postural assessment, static spinal load, and perceived exertion from Borg scale	Medium
				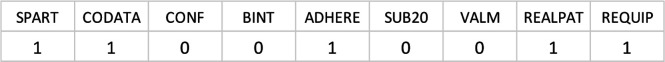	
Jager, Jordan, Theilmeier, Wortmann, Kuhn, Nienhaus, Luttmann	2013	2 female caregivers with extensive professional experience	Germany	*Laboratory study*: Estimated spine load and identified load reduction by applying biomechanically ‘optimized’ transfer modes	Medium
				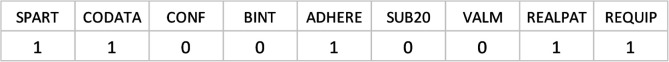	
Katsuhira, Sasaki, Asahara, Ikegami, Ishihara, Kikuchi, Hirai, Yamasaki, Wada, Maruyama	2008	10 male students	Japan	*Laboratory study*: Measured body postures and ground reaction forces and predicted trunk moments during transfers from wheelchair to bed using low back belt on patient, on caregiver, and on both	Medium
				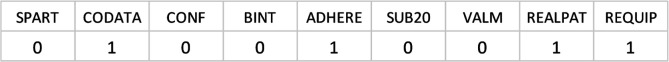	
Keir, MacDonell	2004	7 participants: 4 novice males, 1 experienced female, and 2 experienced males	Canada	*Laboratory Study*: Measured muscle activity patterns during manual transfers and transfers using floor and ceiling lifts	Low-medium
				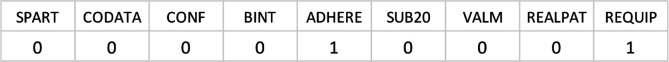	
Koppelaar, Knibbe, Miedema, Burdorf	2012	186 nurses in nursing homes (179 females and 7 males)	The Netherlands	*Field Study*: Direct observation of nurses in nursing homes, quantifying postures and estimating forces	Medium
				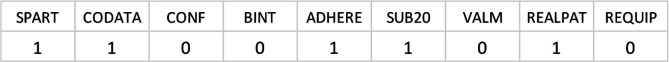	
Kothiyal, Yuen	2004	10 participants	Australia	*Laboratory study*: Measured muscle activity and perceived exertion when conducting patient transfer tasks with and without patient sling assist device	Low-medium
				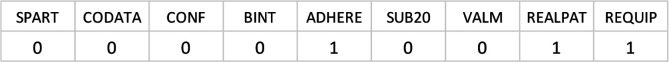	
Kotowski, Davis, Marras	2019	16 participants (8 males and 8 females)	United States	*Laboratory study*: Estimated spine load during repositioning and lateral transferring with 6 devices (draw sheet, reusable air-assisted device, disposable air-assisted device, dual friction-reducing sheets, slide board, and friction-reducing covered board (lateral transfer only)	Medium
				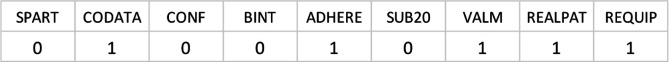	
Law, Ridzwan, Ripin, Hamid, Law, Karunagaran, Cajee	2022	6 nurses with minimum of 6 months experience	Malaysia	*Laboratory study*: Measured full body kinematics, ground reaction forces, and hand forces	Medium
				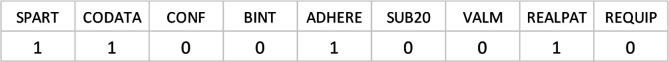	
Lloyd, Baptiste	2006	1 male	United States	*Laboratory study*: Estimated spinal loads and joint moments from 11 different lateral transfer technologies and techniques	Low-medium
				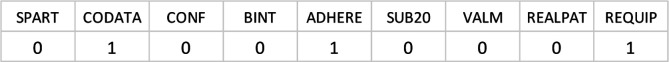	
Marras, Knapik, Ferguson	2009	5 male and 5 female inexperienced students	United States	*Laboratory study*: Estimated spine loads during pushing and pulling with two types of floor and ceiling lifts	Medium
				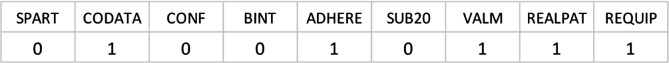	
McGill, Kavcic	2005	3 male subjects trained in patient handling	Canada	*Laboratory study*: Measured muscle activity and applied force, estimated spinal loads using 3 lateral sliding transfer devices	Medium
				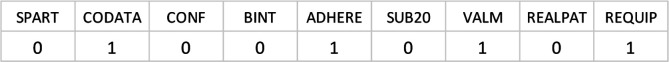	
Muono, Vartiainen, Karjalainen, Räsänen	2022	2 experienced nurses	Finland	*Laboratory study*: Measured ground reaction forces and rating of perceived exertion	Medium
				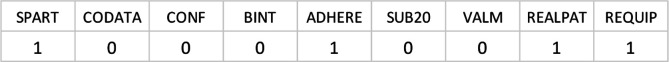	
Nelson, Lloyd, Menzel, Gross	2003	134 nursing personnel (registered nurses, licensed practical nurses, nursing assistants) with 71 in control group and 63 in intervention group	United States	*Laboratory study*: Measured muscle activity, hand forces, and perceived exertion, predicted spine forces and joint moments for interventions for 9 common patient handling tasks	Medium
				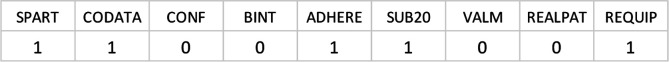	
Nevalaa, Tamminen-Peter	2004	12 healthy female nurses	Finland	*Field study*: Measured muscle activity and muscle strain for adjustable versus traditional shower trolley	Low-medium
				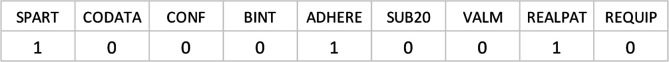	
Nussbaum, Torres	2001	24 female students with no experience in patient handling	United States	*Laboratory study*: Measured perceived exertion and estimated spine loads when performing patient handling tasks with two levels of training	Medium
				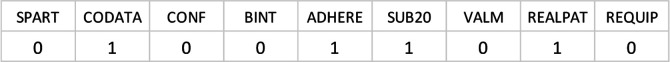	
Omura, Hirati, Yochimine, Nakatani, Inoue	2022	28 clinical nurses and care workers	Japan	*Laboratory study*: Measured trunk flexion and rating of comfort and fatigue	Medium
				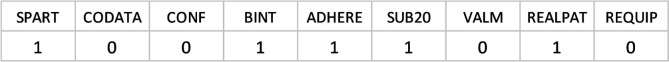	
Potvin	2017	Simulated female (50th percentile)	Canada	*Laboratory study*: Estimated spine compression and shear forces, torques on arms, knee, and hip, and shoulder trunk posture when transferring patient out of recliner, bed, and chair with and without gait belt	Low-medium
				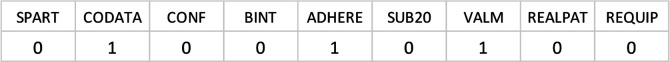	
Riccoboni, Monnet, Eon, Lacouture, Gazeau, Campone	2021	9 caregivers (7 females and 2 males)	France	*Laboratory study*: Measured forces and torques in lumbar spine, perceived exertion, and discomfort when standing patient and sitting patient in chair manually (single and two-person) versus motorized lift	Medium
				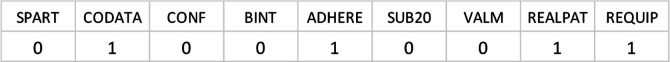	
Rice, Woolley, Waters	2009	1 standard male participant did pushing/pulling	United States	*Laboratory study*: Measured push, pull and rotating forces for lifting devices (floor-based power stand-up lift, floor-based full body lift with a divided-leg sling, and overhead mounted full body lift with a high-back sling	Medium
				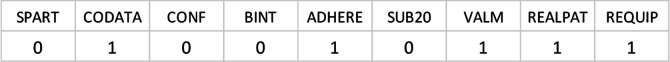	
Santaguida, Pierrynowski, Goldsmith, Fernie	2005	5 female registered nurses	Canada	*Laboratory study*: Predicted spine loads and measured perceived exertions for bed to chair transfer comparing overhead and floor powered lifts	Medium
				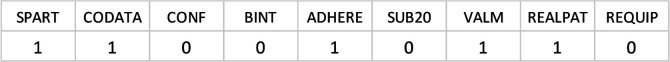	
Schibye, Hansen, Hye-Knudsen, Essendrop, Bocher, Skotte	2003	9 female health care workers	Denmark	*Laboratory study*: Estimated spinal loads and measured perceived exertion for different transfers and devices	Medium
				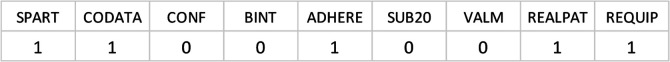	
Silvia, Bloswick, Lillquist, Wallace, Perkins	2002	6 subjects (4 females and 2 males) who were formally trained in patient handling	United States	*Laboratory study*: Estimated spine loads for transfer tasks with and without lift assist devices	Medium
				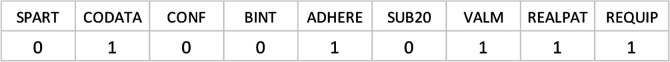	
Tang, Holland, Milbauer, Olson, Skora, Kapellusch, Garg	2018	28 students	United States	*Laboratory study*: Estimated spine loads, strength requirements, and perceived exertion for bed-to-wheelchair transfers with walking belt versus gait belt	Medium
				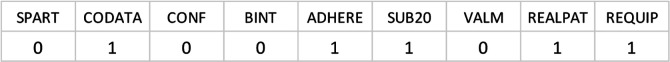	
Theou, Soon, Filek, Brims, Leach-MacLeod, Binsted, Jakobi	2011	5 healthcare providers	Canada	*Laboratory study*: Measured muscle activity for arm and shoulder muscle and assessed rating of perceived exertion	Low-medium
				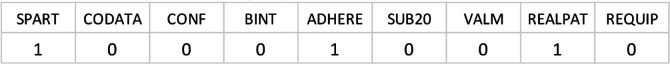	
Ulin, Chaffin, Patellos, Blitz, Emerick, Lundy, Misher	1997	2 nursing students	United States	*Laboratory study*: Estimated spine loads, strength requirements, and hand forces when transferring patients with screw activated lift, hydraulic lift, and electric lift versus manual lifting	Medium
				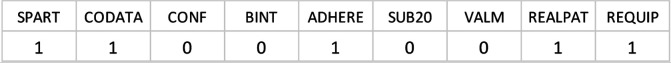	
Vinstrup, Jakobsen, Madeleine, Andersen	2020	52 female healthcare workers from 16 different departments at 5 Danish hospitals	Denmark	*Field study*: Measured muscle activity and trunk posture during a full workday	Medium
				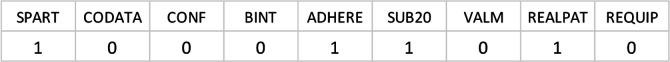	
Walls	2001	1 staff member acted as nurse and 1 staff member acted as patient	New Zealand	*Field study*: Measured trunk kinematics for electrical beds and manual beds	Medium
				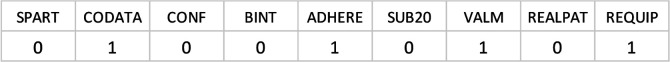	
Waters, Dick, Lowe, Werren, Parsons	2012	1 female operator	United States	*Laboratory study*: Measured hand forces and estimated spinal loading for overhead-mounted lifts and floor-based lifts across various floor surfaces and patient weight conditions	Low-medium
				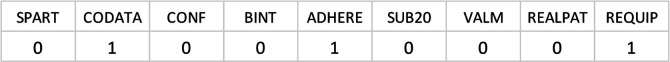	
Weiner, Kalichman, Ribak, Alperovitch-Najenson	2017	12 nurses	Israel	*Laboratory study*: Measured torso kinematics and perceived exertion when performing 27 patient transfers	Medium
				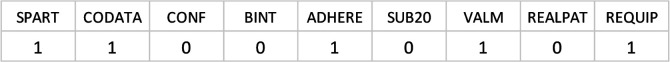	
Wiggermann	2016	9 female nurses	United States	*Laboratory study*: Measured compression, shear forces, hand forces, and shoulder strength for with and without turn assist when turning and laterally repositioning patients	Medium
				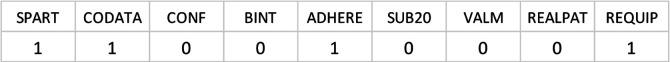	
Wiggermann, Zhou, McGann	2021	10 female participants	United States	*Laboratory study*: Measured hand forces and ground reaction forces to estimate compression spine force for repositioning, turning, and lateral transferring with different lift aides	Medium
				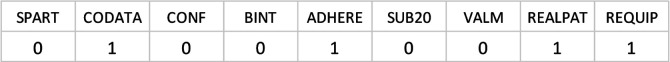	
Wiley	2001	18 female occupational Therapy students	United States	*Laboratory study*: Measured trunk flexion, knee flexion, and elbow flexion for lifting belt versus no belt during patient transfers	Low
				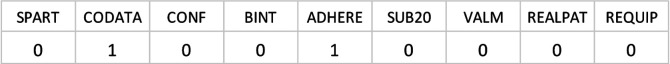	
Zakerian, Afzalinejhad, Mahmodi, Sheibani	2021	60 staff members of the ICU	Iran	*Field study*: Measured rating of perceived exertion	Medium
				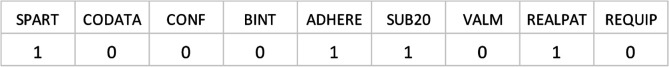	
Zhuang, Stobbe, Hsiao, Collins, Hobbs	1999	9 nursing assistants	United States	*Laboratory study*: Evaluated of nine battery-powered lifts, a sliding board, a walking belt, and a baseline manual method for transferring nursing home residents from a bed to a chair	Medium
				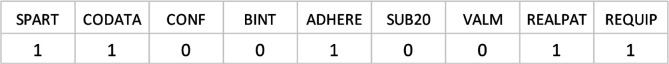	

*Under Study Design, the table provides the assessments for the quality ratings: SPART—participants representative of population, CODATA—complete outcome data, CONF—confounders accounted for, BINT—Outcome assessors blinded to intervention, ADHERE—adherence to intervention, SUB20—sufficient numbers of subjects (*N* >20), VALM—validated measures, REALPAT—realistic patients being moved, and REQUIP—reliable equipment.

**Table 2.** Summary of the Number of Studies That Found a Decrease (↓), No Difference, or Increase (**↑**) Relative to Manual or Draw Sheet and the Various Safe Patient Handling Equipment or Techniques for Each of the Outcomes

	Proper Lifting	Slide Board and Roller Board	Gait belt and Walking Belt	Friction Reducing Sheets and Plastic Bags	Air-Assisted Device	Floor Lifts	Ceiling Lifts
Spine posture		**↓: 1**	No Diff: 1 **↓: 1**	No Diff: 1	**↓: 1**	No Diff: 1	
Motion capture	**↓: 2** No Diff: 1	**↓: 2** No Diff: 2	**↓: 5**	No Diff: 2	**↓: 1**	**↓: 2**	**↓: 1**
Other joint position			No Diff: 1			**↓: 1** No Diff: 1	
Physiological measures			**↑: 1**			No Diff: 1	
Subjective force	**↓: 1**	**↓: 5** No Diff: 5	**↓: 5** No Diff: 1	**↓: 5** No Diff: 1	**↓: 4**	**↓: 5** **↑: 1**	**↓: 2**
Discomfort		**↓: 2**	**↓: 2**	**↓: 2**	**↓: 1**	**↓: 3**	**↓: 1**
Ground reaction force		**↓: 2**	**↓: 1**				
Hand or applied force		**↓: 7** No Diff: 1	**↓: 5** No Diff: 1	**↓: 4** No Diff: 2	**↓: 5**	**↓: 1**	**↓: 3**
Muscle activity	**↓: 1**	**↓: 6** No Diff: 1	**↓: 1** No Diff: 1	**↓: 6** No Diff: 1	**↓: 4**	**↓: 1**	**↓: 4**
Spine loads	**↓: 3**	**↓: 1** No Diff: 3	**↓: 5** No Diff: 3 **↑: 1**	**↓: 5** No Diff: 2	**↓: 5**	**↓: 5**	**↓: 2**
Joint torque	**↓: 3**	**↓: 2** No Diff: 3	**↓: 1** No Diff: 2	**↓: 2** No Diff: 1 **↑: 1**	**↓: 3** No Diff: 1	**↓: 2**	**↓: 1**

### Overview of Included Articles

Table 1 provides a summary of the studies with respect to subject population, country where study was performed, study design, and quality of study rating. Studies have been collected at a consistent pace over the years: 1980 to 1999: 7, 2000 to 2004: 13, 2005 to 2009:8, 2010 to 2014: 8, 2015 to 2019: 8, and 2020 to 2023: 13. Studies were conducted across the world with most being in the United States (27) and Canada (10) followed by The Netherlands (3), Australia (2), Denmark (2), Finland (2), Japan (2), and the rest of the countries had one study (China, Germany, Indonesia, Iran, Israel, Lebanon, Malaysia, New Zealand, United Kingdom). Majority of the studies included in this review were laboratory based (49), while one was both laboratory and field, and the remaining were field (7). The quality assessment yielded one high quality studies, 6 medium-high quality studies, 13 low-medium studies, and 1 low study. The vast majority were rated medium quality (36).

## FINDINGS AND DISCUSSION

Table 2 provides a summary of the results for the different modalities of safe patient handling. One interesting finding was that there are few studies (4) reporting a negative result (e.g., increase in outcome variable) for any safe patient handling modality. This indicates that in the worst case, the patient handling equipment was equivalent to manual lifting. There was one high, six medium-high quality studies, thirty-six medium quality studies, and thirteen low-medium quality studies, and one low quality study.

Proper lifting has been a target for safe patient handling due to being a cheap and easy to implement intervention. The concept is to use proper body mechanics to limit the stress on the body, specifically on the spine, by keeping the patient close, bending the knees, and keeping the back upright. Biomechanical studies have shown a reduction in joint postures—more neutral postures (2 studies), trunk moments (3 studies), and spine loads (3 studies) when using proper lifting as compared to no training. Only one study found no difference in joint postures between proper lifting technique and nontraining lifting. Under controlled laboratory conditions (all have been rated medium quality), proper lifting appears to be effective in reducing the stresses on the body. Basically, healthcare givers were more upright and closer to the patient which reduced the trunk moments, muscle activity, and corresponding spine loads when using “proper lifting technique.” However, caution should be taken as training has limitations in the real world when work demands are high, fatigue sets in, and returning to normal lifting. Most biomechanical laboratory studies utilize a compliant simulated patient that will often minimize the biomechanical responses for the caregivers (Marras et al., 1999). Sudden changes in the patient due to losing balance, sudden strength imbalance, or mental impairment may result in a sudden load on the caregiver who is holding them. Lifting or transferring a noncompliant patient is much different than compliant as sudden movements or dead weight of a patient can produce significant additional force requirements for the caregiver, oftentimes unexpected and rapidly. Further, Nelson and Baptiste (2006) provided significant evidence that proper lifting mechanics and safe lifting techniques are not effective in controlling injuries due to patient handling. The bottom line, there is no way to safely manually handle patients (Nelson et al., 2007; Wilson & Davis, 2016). Finally, there are relatively few biomechanical studies (only medium quality studies) investigating proper lifting training to really form a decisive conclusion on training effectiveness to protect caregivers during patient handling.

Slide boards and roller boards have been found relatively ineffective (no difference) with slightly more studies that found them to reduce the biomechanics as compared to manual lifting (25 reduced vs. 15 no difference) (Table 2). For many of the outcomes, slide/roller boards were found to be equivalent to manual handling. The two biomechanical outcome variables that did see consistent benefit of slide/roller boards were hand forces (7 studies found decrease hand forces, 1 no difference) and muscle activity (6 studies found decrease muscle activity, 1 no difference). These reductions in hand force and muscle activity do not seem to translate to reductions in trunk moments and spine loads. While the friction force between the boards and body are likely reduced, the caregiver must still move significant body weight. Further, the roller board devices are only viable during lateral transfers while slide boards can be utilized in lateral transfers and repositioning. The studies evaluating slide/roller boards were generally medium quality with two being medium-high quality and one high quality. The medium-high and high quality studies found decreases in shoulder and trunk flexion, reduced perceived effort, reduced hand forces, lower muscle activations, and low spine loading for slide board as compared to draw sheet (Budarick et al., 2020; Hwang et al., 2019, 2020). Overall, except for reducing hand forces, slide boards and roller boards appear to be a marginally viable solution in protecting caregivers during handling patients.

The use of a belt (gait or walkingbelts) has had mixed results for biomechanical outcomes. Belts have been shown to reduce nonneutral postures (5 studies) and hand forces (5 studies, 1 no difference), but to a lesser extent, limited impact on spine loads (5 studies decrease, 3 studies no difference, and 1 study increase). There were few studies that were rated medium quality investigating gait/walking belts, thus providing further support that belts have limited utility in patient handling. Studies for gait and walking belts have the same concerns as research for proper lifting technique as noncompliant patients can still pose significant risk for the caregiver. Furthermore, gait and walking belts are predominantly used during transfers of patients (e.g., bed to standing, bed to chair, bed to toilet, and bed to wheelchair) and are not applicable to repositioning and lateral transferring. With about equal as many studies finding no difference or worse results as lifting manually, gait and walking belts do not appear to be viable safe patient handling devices.

Friction reducing sheets or use of plastic bags have similar results to the slide/roller boards where these devices were effective in reducing the hand forces (4 studies found decrease, 2 found no difference) and muscle activity (6 studies found reduced, 1 study with no difference), These changes did appear to reduce the biomechanical loading on the spine (5 studies report decreases and 2 study no difference). Two medium-high quality studies (Hwang et al., 2019, 2020) found lower hand forces, less muscle activity, and lower spine loads for frictionless sheets as compared to manual lifting. Several medium rated quality studies found no difference in friction reducing sheets and manual lifting (Kotowski et al., 2022; Weiner et al., 2017). The friction reducing sliding devices can only be utilized for repositioning and lateral transferring of patients, not actual transfers in and out of bed. Overall, there appears some utility for these devices as they reduce the resistant forces between the bed and patient (e.g., friction and shear forces), ultimately reducing the biomechanical requirements and loads on the caregivers.

Air-assisted devices have been shown to be effective in reducing the biomechanical stress on the caregivers but are restricted to repositioning and lateral transfers. All but one study (trunk moments) revealed reductions in the outcomes when compared to manual handling (with decreases in 2 studies for postures, 5 studies for hand forces, 4 studies for muscle activity, 5 studies for spine loading, and 3 studies for trunk moments). Most of the studies investigating air-assisted devices were rated medium or better (Hwang et al., 2019, 2020; Kotowski et al., 2022; Omura et al., 2022; Wiggermann et al., 2021) and yielded the positive results. These devices provide an air cushion between the body and bed/stretcher that allows the caregiver to easily slide the patient over the bed with minimal effort. One concern for these devices is the need to place the air mattress under the patient, which requires additional movement and handling (e.g., need to roll and place under patient and requiring manual movement of patient) or placing the air mattress under the patient when arriving at the facility and leaving under until needed. One concern with these air mattresses under the patient for long-term periods is concern for infection and bacteria control. The air mattress studies did not evaluate the biomechanical responses during the positioning of the air mattresses under the patient, but rather focused on the transfer and repositioning tasks.

Mechanical lifts, floor and ceiling, were found to be effective in reducing many of the biomechanical outcomes with few “no difference” outcomes (2 studies relating to positional outcomes) (see Table 2). All studies that investigated ceiling lifts found decreases in biomechanical outcomes, by far the most effective patient handling device. Most studies that compared floor lifts to ceiling lifts found ceiling lifts to be superior in reducing spine loads and trunk moments as well as other biomechanical outcomes (Dutta et al., 2011, 2012; Marras et al., 2009; Santaguida et al., 2005; Waters et al., 2012). Ceiling lifts have also been found to be the preferred method for reducing actual injuries (Asuquo et al., 2021; Chhokar et al., 2005; Engst et al., 2005; Lee & Rempel, 2020; Villeneuve, 1998). Overall, mechanical lift studies were rated at medium or better quality and found lifts to be effective in reducing biomechanical loading, for all types of patient handling: transfers, repositions, and lateral transfers.

The review provides insight into the benefit of lifting devices as it was apparent that manual lifting was not safe, no matter how good your proper lift mechanics. Based on the current evidence, the best way to protect healthcare workers is to utilize lifts, preferably ceiling lifts.

### Missing Evidence

There are several shortcomings of the current studies. First, there are several biomechanical outcomes that have had limited investigation. Most safe patient handling interventions have had limited physiological assessment that will provide evaluation of fatigue. Additionally, for any one intervention modality and specific biomechanical outcome, few studies were found (at most 7 studies) with most of these being rated medium-low to low quality (40 out of 56 studies, 71%). Second, all of the studies evaluated the patient handling intervention have concentrated on transfers (e.g., from bed), repositioning, and lateral transfers. Other handling tasks such as sit-to-stand, turning patient in bed, limb lifting, and repositioning and some more high hazard activities like supporting people with limited balance and those that fall need to be investigated with respect to biomechanical outcomes. Third, there is a need for more comprehensive investigations that include basic biomechanical outcomes (e.g., hand forces, positions, and muscle activity) as well as more complex outcomes (e.g., joint moments and spine loads) as many of the studies relied on a single simple assessment. Fourth, one of the most disturbing results was that very few studies investigate nonnursing healthcare workers. With the focus on mobility now being undertaken by physical therapists (PT) or occupational therapists (OT), future studies need to understand the differences in patient handling demands for PT/OT versus nurses. Thus, the current review provides a plethora of evidence about many patient handling equipment but there remains a tremendous amount of future work that needs to be done for a more complete picture.

### Limitations of the Review

There are several potential imitations that should be considered with the current review. First, the review only covers articles published in English. While most journals fall into this category, several additional studies may have been overlooked. Second, biomechanical outcomes are only one type of outcome that relates to safe patient handling. Other factors such as patient safety, patient comfort, risk of injury, cognitive demands, and time to complete the handling task should be reviewed for a more comprehensive understanding of patient handling devices. Finally, most of the studies included in this review were rated below high quality, which limits the ultimate understanding of the effectiveness of patient handling devices. Some people feel reviews should only contain the highest quality, but we wanted to include all biomechanical studies. Future research should strive to have high quality by including well-validated measures such as complex spine loading models, recruit actual healthcare workers for participants with large numbers, use an actual person simulating a noncompliant patient, and use a strong study design.

## Conclusion

Current best practice guidelines adopted by many international governmental systems suggest that the use of assistive devices is an essential step to risk reduction for care workers. This review shows an increase in the volume of studies and higher quality evidence that the use of assistive devices does indeed reduce the physical loads on caregivers. This cumulative knowledge should support the increased use of suitable assistive devices in care delivery. Some areas of patient handling still require further investigation to further reduce the overload of care workers form specific transfers such as a sit-to-stand, walking and falling patients, and other postural static loads that are evident in care work.

## APPENDIX

**Table A1.** Summary of the Quantitative and Subjective Results for Safe Patient Handling Interventions.

Author	Spine Posture	Motion Capture	Other Joint Position	Physiological Measures	Subjective Force	Discomfort
Al-Qaisi et al., 2020					↓ effort when OR table tilted versus flat; ↓ effort when using plastic bag and slide board versus sheet	
Bacharach et al., 2016					↓ effort to move using the nylon tube, roller board, and roller tray compared with the cotton sheet and slide board versus sheet	
Cheung et al., 2020	No difference between gait belt and no gait belt				↓ effort for gait belt versus no gait belt	
Drew et al., 2015					↓ effort for slide sheet and modified slide sheet versus sheet	
Dutta et al., 2012					↓ effort for ceiling lift versus floor lift	
Elford et al., 2000	↓ 3D trunk postures and velocities for PH sling versus no sling					↓ discomfort for most body parts for PH sling versus no sling
Gagnon et al., 1986				↑ work and energy for gait belt versus no gait belt		
Garg & Owen, 1992						↓ lift versus walking belt for shoulder, low back, and upper back discomfort
Garg & Owen, 1994		↓ sling, gait and walking belts versus 2-person manual for lateral bend and twist of trunk			↓ sling and gait walking belts versus 2-person manual for perceived stress; j ↓ lift versus gait belt, sling, and 2-person manual for perceived stress	
Garg et al., 1991a		↓ sling and gait and walking belts versus 2-person manual for lateral bend of trunk			↓ perceived stress for sling and walking belt versus 2-person manual for perceived stress; ↓ lift versus gait belt, sling, and 2-person manual; no difference for perceived stress for Hoyer lift versus two-person manual	↓ lifts, sling, and gait and walking belt versus 2-person manual for discomfort
Garg et al., 1991b		No difference between sling and gait walking belts versus 2-person manual for trunk posture			↓ lift, sling and walking belt versus 2-person manual for perceived stress; ↑ Hoyer lift versus walking belt, sling, and 2-person manual for perceived stress	↓ lifts, sling and walking belt versus 2-person manual for discomfort
Hess et al., 2007	No LBD risk between pivot, scoot, and slide board transfer to wheelchair; ↓ sagittal flexion for slide and pivot versus scoot; ↓ lateral flexion for slide and scoot versus pivot; ↓ twist for pivot versus scoot and slide				No difference for RPE between pivot, scoot, and slide board transfer to wheelchair	
Hodder et al., 2010		↓ sagittal flexion and lateral bend between trained (proper lifting techniques) versus untrained for repositioning; No difference for transfer from bed to wheelchair				
Hwang et al., 2020		↓ trunk flexion for air-assisted device, slide board, and frictionless sheet versus no device for turning towards and away tasks; ↓ shoulder flexion for air-assisted device versus no device for turning towards task			↓ effort of use for air-assisted device versus no device for turning towards and away tasks	
Hwang et al., 2019		↓ shoulder flexion and abduction for air-assisted device versus slide board, draw sheet and frictionless sheet for lateral transfers and repositioning; ↓ trunk flexion and abduction for air-assisted device versus slide board, draw sheet and frictionless sheet for lateral transfers and repositioning			↓ effort to use for air-assisted device versus slide board, draw sheet and frictionless sheet for lateral transfers and repositioning	
Iridiastadi et al., 2020					↓ effort with floor lift as compared to manual	
Katsuhira et al., 2008		↓ sagittal trunk flexion when wearing back belt versus no back belt during seated transfer				
Koppelaar et al., 2012			↓ nonneutral trunk posture for lift device versus no lift device for repositioning; no significant difference for transfer			
Kothiyal & Yuen, 2004					↑ perceived effort in low back and shoulders for gait belt versus no gait belt	
Kotowski et al., 2022					↓ perceived exertion with air-assisted device versus slide board, dual friction sheets and draw sheet for repositioning; ↓ perceived exertion with air-assisted device versus slide board, friction reducing board, dual friction sheets and draw sheet for lateral transfers; manual lifting had highest	↓ discomfort with air-assisted device versus slide board, dual friction sheets and draw sheet for repositioning; ↓ discomfort with air-assisted device versus slide board, friction reducing board, dual friction sheets and draw sheet for lateral transfers; manual lifting had highest
Muona et al., 2022					↓ perceived exertion with slide film as compared to regular sheet	
Nelson et al., 2003						↓ discomfort with ceiling mounted lift, friction reducing device, and stretcher that converts to a chair versus manual for transfer tasks
Nevala & Tamminen-Peter, 2004				No difference in heart rate electrically adjustable with shower trolley versus traditional shower	↓ perceived strain in neck/shoulder, back, arms, and legs with electric trolley versus traditional show for shower tasks	
Nussbaum & Torres, 2001		↓ knee, elbow, and trunk angles for training in good lifting versus none			↓ perceived exertion in whole body, shoulder, low back for training in good lifting versus none	
Omura et al., 2022	↓ spine flexion angle for air-assisted turn device compared to manual				↓ perceived fatigue for air assisted turn device compared to manual	
Silvia et al., 2002					↓ effort to use for Barton System versus 2-person draw sheet for bed to chair and chair to bed transfers; ↓ effort to use for Barton System versus 2-person draw sheet for lateral transfers	
Santaguida et al., 2005					↓ perceived effort with ceiling lift versus floor lift for bed to wheelchair transfers	
Tang et al., 2018		↓ trunk flexion for gait belt versus walking belt for males; ↓ lateral flexion for walking belt versus gait belt for males and females			↓ perceived effort for walking belt versus gait belt	
Theou et al., 2011					↓ perceived effort with slider system versus traditional bed sheet	
Ulin et al., 1997					↓ perceived effort for mechanical assist device (screw-activated, hydraulic, and electric) versus manual (pivot, slide board, and gait belt) for patient transfers	
Vinstrup et al., 2020		↓ trunk flexion with ceiling lifts and intelligent beds versus manual, draw sheet, sliding sheet, and sliding boards during patient transfers; ↓ lateral bending with ceiling lifts versus manual, intelligent beds, draw sheet, sliding sheet, and sliding boards during patient transfers				
Walls, 2001	No difference between electric bed versus manual bed for LBD risk					
Weiner, et al., 2017	No difference between sliding sheet, lifting hoist, and draw sheet for LBD risk for repositioning				↓ perceived effort with lift hoist and sliding sheet versus draw sheet for bed to wheelchair transfers for repositioning	
Willey, 2001		↓ sagittal trunk flexion when wearing back belt versus no back belt during patient transfer; No difference between belt and no belt for elbow and knee position				
Zakerian et al., 2021					↓ perceived effort with lift belt versus manual	↓ discomfort in neck, shoulders and arms, waist, hands and wrists, buttocks, thighs, knees and legs. With lift belt versus manual No difference in discomfort in elbow and forearm between lift belt and manual
Zhuang et al., 1999		↓ sagittal flexion, lateral flexion, and twist for trunk when placing sling and using ceiling lift versus manual with gait belt when positioning for transfer; ↓ sagittal flexion, lateral flexion, and twist for trunk when stand-up lift and sliding board versus manual with gait belt when positioning for transfer				

**Table A2.** Summary of the Biomechanical Loading Results for Safe Patient Handling Interventions.

Author	Ground Reaction Force	Hand or Applied Force	Muscle Activity	Spine Loads	Joint Torque
Allen et al., 2002				No difference in compression for belts, turntables, slings, transfer boards versus no equipment	
Al-Qaisi et al., 2020			↓ trapezius and erector spinae muscle activity when OR table tilted versus flat; ↓ trapezius and erector spinae muscle activity when using plastic bag and slide board versus sheet		
Bacharach et al., 2016		↓ applied hand force using the nylon tube, roller board, and roller tray compared with the cotton sheet and slide board versus sheet			↓ trunk moment using the nylon tube, roller board, and roller tray compared with the cotton sheet and slide board versus sheet
Bartnik & Rice, 2008		No difference in applied hand force between sheet and MaxiSlide and fabric slide sheets		↓ compression, lateral shear, and A-P shear for MaxiSlide and fabric slide sheets versus sheet	
Blaauw et al., 2021			Mixed results with few consistent statistical trends between Strong Arm versus Hoyer Lift		
Budarick et al., 2020		↓ hand forces (to none) when using turn assist compared to manual		↓ compression when using turn assist compared to manual	↓ shoulder moments when using turn assist compared to manual
Cheung et al., 2020			↓ one muscle (RES) for gait belt versus no belt, no difference in rest		
Daynard et al., 2001		↓ applied hand force for friction reducing devices during boost in bed		↓ compression for friction reducing devices during boost in bed; ↓ compression for gait belt and even more with mechanical lift; few differences in cumulative compression and shear	
Drew et al., 2015		↓ applied hand force for slide sheet and modified slide sheet versus sheet	↓ muscle activity for erector spinae for slide sheet and modified slide sheet versus sheet		
Dutta et al., 2011				↓ external forces and moments at L5/S1 for ceiling lift versus floor lift	
Dutta et al., 2012				↓ external forces and moments at L5/S1 for ceiling lift versus floor lift	
Gagnon et al., 1986				↑ compression for gait belt versus no gait belt	
Garg & Owen, 1992		↓ hand forces with walking belt versus manual		↓ compression with walking belts versus manual	
Garg & Owen, 1994		↓ hand forces sling and gait and walking belts versus 2-person manual		↓ compression and A-P shear for sling and gait and walking belts versus 2-person manual	
Garg et al., 1991a		↓ hand forces with gait and walking belts versus manual		↓ compression and A-P shear for sling and gait and walking belts versus 2-person manual	
Garg et al., 1991b		↓ hand forces with gait and walking belts versus manual		↓ compression and A-P shear for sling and gait and walking belts versus 2-person manual	
Grevelding & Bohannon, 2001		↓ hand forces with sliding tube and fabric tube versus manual			
Hess et al., 2007		↓ hand force for slide versus scoot and pivot when transferring to wheelchair			
Hodder et al., 2010			↓ muscle activity between trained (proper lifting techniques) versus untrained for trapezius, erector spinae and external oblique for repositioning and transfer to wheelchair		
Howard et al., 2013			↓ muscle activity in Biceps, extensors, and erector spinae for ceiling lift versus manual, scoot, and modified scoot		
Hwang et al., 2020			↓ muscle activity in all seven muscles for air-assisted device, slide board, draw sheet and frictionless sheet versus no device for turning towards and away tasks		↓ flexion moment for air-assisted device versus no device for turning towards and away tasks; ↑ extension moment for friction-reducing turning sheet versus no device for turning towards and away tasks
Hwang et al., 2019		↓ hand forces with slide board and air-assisted device versus draw sheet and frictionless sheet for lateral transfers	↓ muscle activity in all seven muscles for air-assisted device versus slide board, draw sheet and frictionless sheet for lateral transfers and repositioning		↓ shoulder moment for air-assisted device versus slide board, draw sheet and frictionless sheet for lateral transfers and repositioning
Iridiastadi et al., 2020				↓ compression force with floor lift as compared to manual ↑ small amount of shear force with floor lift as compared to manual	
Jäger et al., 2013				↓ compression loads using proper lifting versus no training lifting repositioning in bed and transfer to chair; ↓ compression loads using slide aide versus proper lifting repositioning in bed and transfer to chair	↓ torsional and lateral moments using proper lifting versus no training lifting transfer to chair; ↓ torsional and lateral moments using slide aide versus proper lifting repositioning in bed and transfer to chair
Katsuhira et al., 2008	↓ vertical ground reaction force when wearing back belt versus no back belt during seated transfer				↓ sagittal and lateral trunk moment when wearing back belt versus no back belt during seated transfer
Keir & MacDonell, 2004			↓ muscle activity in all eight muscles ceiling lift versus floor lift and manual lifting; ↓ muscle activity in all eight muscles floor lift versus manual lifting for bed to wheel chair and wheelchair to bed		
Koppelaar et al., 2011		↓ exerted forces for lift device versus no lift device for repositioning and transfer			
Kothiyal & Yuen, 2004			↑ muscle activity for erector spinae for sling device versus manual		
Kotowski et al., 2022			↓ muscle activity in the latissimus dorsi, rectus abdominus, and external oblique with air-assisted device versus draw sheet for repositioning and lateral transfers	↓ lateral shear and compression with air-assisted device versus slide board, dual friction sheets and draw sheet for repositioning; ↓ A-P shear, lateral shear and compression with air-assisted device versus slide board, friction reducing board, dual friction sheets and draw sheet for lateral transfers	
Law et al., 2022	↓ external forces with sliding board as compared to manual	↓ hand forces with sliding board reduced as compared to manual			
Lloyd & Baptiste, 2006		↓ hand force with air-assisted device, lateral transfer device, slide boards, and frictionless sheet set versus draw sheet for lateral transfer tasks		↓ compression and A-P shear with air-assisted device, lateral transfer device, and frictionless sheet set versus draw sheet for lateral transfer tasks	↓ shoulder, elbow, and torso moments with air-assisted device, slide boards, lateral transfer device, and frictionless sheet set versus draw sheet for lateral transfer tasks
Marras et al., 2009				↓ A-P shear, lateral shear and compression with ceiling lift versus floor lift for patient transfer in room	
McGill & Kavcic, 2005		↓ hand force with friction reducing covered board versus bed sheet during pushing and pulling lateral transfer	↓ extensor muscles with friction reducing covered board versus bed sheet during pushing and pulling lateral transfer	↓ A-P shear with friction reducing covered board versus bed sheet during pushing and pulling lateral transfer	
Muona et al., 2022	↓ ground reaction forces with slide film as compared to regular sheet				
Nelson et al., 2003			↓ muscle activity in shoulder and erector spinae with ceiling mounted lift, friction reducing device and stretcher that converts to a chair versus manual for transfer tasks	↓ compression force with ceiling mounted lift, friction reducing device, and stretcher that converts to a chair versus manual for transfer tasks	↓ shoulder and low back moments with ceiling mounted lift, friction reducing device and stretcher that converts to a chair versus manual for transfer tasks
Nevala & Tamminen-Peter, 2004			↓ muscle activity in erector spinae and trapezius with electrically adjustable shower trolley versus traditional shower trolley for shower tasks		
Nussbaum & Torres, 2001				↓ compression and A-P shear for training in proper lifting versus none	↓ low back moment for training in proper lifting versus none
Potvin, 2017		No difference hand force between gait belt and no gait belt		No difference compression and shear between gait belt and no gait belt	No difference hip and knee moment between gait belt and no gait belt
Rice et al., 2009		↓ hand force with ceiling lift versus floor lift and stand-up lift for pushing and pulling of lifts			
Riccoboni et al., 2021				↓ compression with motorless assisted device versus manual handling with 1 and 2 people during sitting and standing of person	↓ low back moment with motorless assisted device versus manual handling with 1 and 2 people during sitting and standing of person
Santaguida et al., 2005				↓ A-P shear and compression with ceiling lift versus floor lift for bed to wheelchair transfers	
Schibye et al., 2003				↓ compression for training in proper lifting versus none for repositioning, elevate to seated position, bed to standing, and reposition in wheelchair; ↓ A-P shear for training in proper lifting versus none for turning, repositioning, elevate to seated position, and bed to standing	↓ trunk moment for training in proper lifting versus none for repositioning, elevate to seated position, bed to standing, and reposition in wheelchair
Silvia et al., 2002				↓ compression for Barton System versus 2-person draw sheet in lateral transfers; ↓ compression for Barton System and mechanical lift versus 1-person hug in bed to chair transfer; ↓ compression for Barton System versus 2-person and 1-person hug and 2-person draw sheet in chair to bed transfer; ↓ compression for Barton System versus 2-person (hug and 1-person hug and 2-person draw sheet in repositioning	
Tang et al., 2018		↓ applied hand force for walking belt versus gait belt when transferring patient for males and females		↓ compression and A-P shear at L5/S1 for walking belt versus gait belt when transferring patient for males and females; ↓ compression at L4/L5 for walking belt versus gait belt when transferring patient for males and females; ↓ A-P shear at L4/L5 for gait belt versus walking belt when transferring patient for males and females	↓ elbow, shoulder, and trunk moment for walking belt versus gait belt when transferring patient for males and females
Theou et al., 2011			↓ number and duration of EMG bursts with slider system versus traditional bed sheet		
Ulin et al., 1997				↓ compression for mechanical assist device (screw-activated, hydraulic, and electric) versus manual (pivot, slide board, and gait belt) for patient transfers	↓ shoulder moment for mechanical assist device (screw-activated, hydraulic, and electric) versus manual (pivot, slide board, and gait belt) for patient transfers
Vinstrup, et al., 2020			↓ muscle activity in erector spinae with ceiling lifts and intelligent beds versus manual, draw sheet, sliding sheet, and sliding boards during patient transfers		
Waters et al., 2012		↓ hand forces with ceiling lift versus floor lift for patient transfers with larger differences for carpet		↓ compression with ceiling lift versus floor lift for patient transfers with larger differences for carpet	
Wiggermann, 2016		↓ hand forces with turn assist versus manual turning and lateral repositioning of patient in bed		↓ compression and A-P shear with turn assist versus manual turning and lateral repositioning of patient in bed	No difference in shoulder moment for turn assist versus manual turning and lateral repositioning of patient in bed
Wiggermann et al., 2021		↓ hand forces with air-assisted device and friction reducing sheets versus draw sheet and glide sheet during repositioning; ↓ hand forces with air-assisted device and friction reducing sheets versus draw sheet during lateral transfer; ↓ hand forces with turn assisted device versus manual during turning	↓ muscle activity in brachialis with air-assisted device and friction reducing sheets versus draw sheet and glide sheet during repositioning	↓ compression with air-assisted device versus draw sheet, friction reducing sheets, and glide sheet during repositioning; ↓ compression with air-assisted device and friction reducing sheets versus draw sheet during lateral transfer	
Zhuang et al., 1999		↓ hand force when placing sling and using ceiling lift versus manual with gait belt when positioning for transfer; ↑ hand force for trunk when stand-up lift and sliding board versus manual with gait belt when positioning for transfer		↓ compression when placing sling and using ceiling lift versus manual with gait belt when positioning for transfer	

## References

[bibr1-00187208231211842] Al Johani W. A. Pascua G. P. (2019). Impacts of manual handling training and lifting devices on risks of back pain among nurses: An integrative literature review. Nurse Media Journal of Nursing, 9(2), 210–230. 10.14710/nmjn.v9i2.26435

[bibr85-00187208231211842] Alamgir H. Li O. W. Yu S. Gorman E. Fast C. Kidd C. (2009). Evaluation of ceiling lifts: Transfer time, patient comfort and staff perceptions. Injury, 40(9), 987–992. 10.1016/j.injury.2008.12.002. 19486965

[bibr2-00187208231211842] Allen R. Jackson S. Marsden H. McLellan D. L. Gore S. (2002). Transferring people safely with manual handling equipment. Clinical Rehabilitation, 16(3), 329–337. 10.1191/0269215502cr499oa 12017520

[bibr3-00187208231211842] Al-Qaisi S. K. El Tannir A. Younan L. A. Kaddoum R. N. (2020). An ergonomic assessment of using laterally-tilting operating room tables and friction reducing devices for patient lateral transfers. Applied Ergonomics, 87, 103122. 10.1016/j.apergo.2020.103122 32501251

[bibr4-00187208231211842] Andersen L. L. Burdorf A. Fallentin N. Persson R. Jakobsen M. D. Mortensen O. S. Clausen T. Holtermann A. (2013). Patient transfers and assistive devices: Prospective cohort study on the risk for occupational back injury among healthcare workers. Scandinavian Journal of Work, Environment & Health, 40(1), 74–81. 10.5271/sjweh.3382 24030699

[bibr5-00187208231211842] Asuquo E. G. Tighe S. M. Bradshaw C. (2021). Interventions to reduce work-related musculoskeletal disorders among healthcare staff in nursing homes; an integrative literature review. International Journal of Nursing Studies Advances, 3, 100033. 10.1016/j.ijnsa.2021.100033 38746711 PMC11080355

[bibr6-00187208231211842] Bacharach D. W. Miller K. von Duvillard S. P. (2016). Saving your back: How do horizontal patient transfer devices stack up? Nursing, 46(1), 59–64. 10.1097/01.NURSE.0000475501.70596.2b 26692313

[bibr7-00187208231211842] Bartnik L. M. Rice M. S. (2008). Comparison of caregiver forces required for sliding a patient up in bed using an array of slide sheets. Workplace Health & Safety, 61(9), 393–400. 10.1177/216507991306100904 23957831

[bibr8-00187208231211842] Blaauw E. R. Greenhalgh M. Vegter R. Bass S. Kulich H. Grindle G. G. Cooper R. Koontz A. M. Cooper R. A. Cooper R. A. (2021). Assessment of muscle activation of caregivers performing dependent transfers with a novel robotic-assisted transfer device compared with the Hoyer advance. American Journal of Physical Medicine & Rehabilitation, 100(9), 885–894. 10.1097/PHM.0000000000001665 33315611

[bibr9-00187208231211842] Bos E. H. Krol B. Van Der Star A. Groothoff J. W. (2006). The effects of occupational interventions on reduction of musculoskeletal symptoms in the nursing profession. Ergonomics, 49(7), 706–723. 10.1080/00140130600578005 16720530

[bibr10-00187208231211842] Budarick A. R. Lad U. Fischer S. L. (2020). Can the use of turn-assist surfaces reduce the physical burden on caregivers when performing patient turning? Human Factors, 62(1), 77–92. 10.1177/0018720819845746 31084493

[bibr11-00187208231211842] Cheung K. Dai J. Cheung C. L. Cho H. K. Chow Y. L. Fung K. Y. Lam W. S. Calvin Li H. L. Ying Ng S. Ngan M. Y. Szeto G. (2020). The biomechanical evaluation of patient transfer tasks by female nursing students: With and without a transfer belt. Applied Ergonomics, 82, 102940. 10.1016/j.apergo.2019.102940 31473499

[bibr12-00187208231211842] Chhokar R. Engst C. Miller A. Robinson D. Tate R. B. Yassi A. (2005). The three-year economic benefits of a ceiling lift intervention aimed to reduce healthcare worker injuries. Applied Ergonomics, 36(2), 223–229. 10.1016/j.apergo.2004.10.008 15694077

[bibr13-00187208231211842] Croshaw C. Fray M. (Eds.), (2018). An illustrated guide to moving and handling people (3rd ed.). Clinical Skills Limited UK. www.clinicalskills.net

[bibr84-00187208231211842] Davis K. G. Kotowski S. E. (2015). Prevalence of musculoskeletal disorders for nurses in hospitals, long-term care facilities and home healthcare: A comprehensive review. Human Factors, 57(5), 754–792. 10.1177/0018720815581933. 25899249

[bibr14-00187208231211842] Dawson A. P. McLennan S. N. Schiller S. D. Jull G. A. Hodges P. W. Stewart S. (2007). Interventions to prevent back pain and back injury in nurses: A systematic review. Occupational and Environmental Medicine, 64(10), 642–650. 10.1136/oem.2006.030643 17522134 PMC2078392

[bibr15-00187208231211842] Daynard D. Yassi A. Cooper J. E. Tate R. Norman R. Wells R. (2001). Biomechanical analysis of peak and cumulative spinal loads during simulated patient-handling activities: A substudy of a randomized controlled trial to prevent lift and transfer injury of health care workers. Applied Ergonomics, 32(3), 199–214. 10.1016/S0003-6870(00)00070-3 11394461

[bibr16-00187208231211842] Drew K. E. Kozey J. W. Moreside J. M. (2015). Biomechanical evaluation and perceived exertion of a lateral patient-handling task. Occupational Ergonomics, 12(4), 151–163. 10.3233/OER-160233

[bibr17-00187208231211842] Dutta T. Holliday P. J. Gorski S. M. Baharvandy M. S. Fernie G. R. (2012). A biomechanical assessment of floor and overhead lifts using one or two caregivers for patient transfers. Applied Ergonomics, 43(3), 521–531. 10.1016/j.apergo.2011.08.006 21875699

[bibr18-00187208231211842] Dutta T. Holliday P. J. Gorski S. M. Baharvandy M. S. Fernie G. R. (2011). The effects of caregiver experience on low back loads during floor and overhead lift maneuvering activities. International Journal of Industrial Ergonomics, 41(6), 653–660. 10.1016/j.ergon.2011.08.003

[bibr19-00187208231211842] Elford W. Straker L. Strauss G. (2000). Patient handling with and without slings: An analysis of the risk of injury to the lumbar spine. Applied Ergonomics, 31(2), 185–200. 10.1016/S0003-6870(99)00026-5 10711981

[bibr20-00187208231211842] Engst C. Chhokar R. Miller A. Tate R. B. Yassi A. (2005). Effectiveness of overhead lifting devices in reducing the risk of injury to care staff in extended care facilities. Ergonomics, 48(2), 187–199. 10.1080/00140130412331290826 15764316

[bibr21-00187208231211842] Gagnon M. Sicard C. Sirois J. P. (1986). Evaluation of forces on the lumbo-sacral joint and assessment of work and energy transfers in nursing aides lifting patients. Ergonomics, 29(3), 407–421. 10.1080/00140138608968274 3698968

[bibr22-00187208231211842] Garg A. Owen B. (1992). Reducing back stress to nursing personnel: An ergonomic intervention in a nursing home. Ergonomics, 35(11), 1353–1375. 10.1080/00140139208967398 1425566

[bibr23-00187208231211842] Garg A. Owen B. (1994). Prevention of back injuries in healthcare workers. International Journal of Industrial Ergonomics, 14(4), 315–331. 10.1016/0169-8141(94)90020-5

[bibr24-00187208231211842] Garg A. Owen B. Beller D. Banaag J. (1991a). A biomechanical and ergonomic evaluation of patient transferring tasks: Bed to wheelchair and wheelchair to bed. Ergonomics, 34(3), 289–312. 10.1080/00140139108967314 1829037

[bibr25-00187208231211842] Garg A. Owen B. Beller D. Banaag J. (1991b). A biomechanical and ergonomic evaluation of patient transferring tasks: Wheelchair to shower chair and shower chair to wheelchair. Ergonomics, 34(4), 407–419. 10.1080/00140139108967325 1860461

[bibr26-00187208231211842] Grevelding P. Bohannon R. W. (2001). Reduced push forces accompany device use during sliding transfers of seated subjects. Journal of rehabilitation research and development, 38(1), 135–139. 11322467

[bibr89-00187208231211842] Hess J. A. Kincl L. D. Mandeville D. S. (2007). Comparison of three single-person manual patient techniques for bed-to-wheelchair transfers. Home Healthcare Now, 25(9), 577–579. 10.1097/01.NHH.0000296114.33696.e5. 18049253

[bibr27-00187208231211842] Hignett S. Crumpton E. Alexander P. Ruszala S. Fray M. Fletcher B. (2003). Evidence-based patient handling: Tasks, equipment and interventions. Routledge. 10.7748/ns2003.04.17.33.33.c3383 12744122

[bibr28-00187208231211842] Hodder J. N. MacKinnon S. N. Ralhan A. Keir P. J. (2010). Effects of training and experience on patient transfer biomechanics. International Journal of Industrial Ergonomics, 40(3), 282–288. 10.1016/j.ergon.2010.01.007

[bibr29-00187208231211842] Howard N. l. Bao S. Kim H. Silverstein B. (2013). Comparison of muscle activity of four types of bed-to-wheelchair transfers. American Journal of Safe Patient Handling & Mobility, 3(14), 16–29.

[bibr30-00187208231211842] Hwang J. Ari H. Matoo M. Chen J. Kim J. H. (2020). Air-assisted devices reduce biomechanical loading in the low back and upper extremities during patient turning tasks. Applied Ergonomics, 87, 103121. 10.1016/j.apergo.2020.103121 32501250

[bibr31-00187208231211842] Hwang J. Kuppam V. A. Chodraju S. S. R. Chen J. Kim J. H. (2019). Commercially available friction-reducing patient-transfer devices reduce biomechanical stresses on caregivers’ upper extremities and low back. Human Factors, 61(7), 1125–1140. 10.1177/0018720819827208 30794442

[bibr32-00187208231211842] Iridiastadi H. Vani T. Yamin P. A. R. (2020). Biomechanical evaluation of a patient-handling technology prototype. International Journal of Technology, 11(1), 180–189. 10.14716/ijtech.v11i1.1332

[bibr33-00187208231211842] Jäger M. Jordan C. Theilmeier A. Wortmann N. Kuhn S. Nienhaus A. Luttmann A. (2013). Lumbar-load analysis of manual patient-handling activities for biomechanical overload prevention among healthcare workers. Annals of Occupational Hygiene, 57(4), 528–544. 10.1093/annhyg/mes088 23253360

[bibr34-00187208231211842] Katsuhira J. Sasaki H. Asahara S. Ikegami T. Ishihara H. Kikuchi T. Hirai Y. Yamasaki Y. Wada T. Maruyama H. (2008). Comparison of low back joint moment using a dynamic 3D biomechanical model in different transferring tasks wearing low back belt. Gait & Posture, 28(2), 258–264. 10.1016/j.gaitpost.2007.12.070 18280736

[bibr35-00187208231211842] Keir P. J. MacDonell C. W. (2004). Muscle activity during patient transfers: A preliminary study on the influence of lift assists and experience. Ergonomics, 47(3), 296–306. 10.1080/0014013032000157922 14668163

[bibr36-00187208231211842] Koppelaar E. Knibbe H. J. Miedema H. S. Burdorf A. (2012). The influence of ergonomic devices on mechanical load during patient handling activities in nursing homes. Annals of Occupational Hygiene, 56(6), 708–718. 10.1093/annhyg/mes009 22393034

[bibr37-00187208231211842] Koppelaar E. Knibbe J. J. Miedema H. S. Burdorf A. (2011). Individual and organisational determinants of use of ergonomic devices in healthcare. Occupational and Environmental Medicine, 68(9), 659–665. 10.1136/oem.2010.055939 21098827 PMC3158329

[bibr38-00187208231211842] Kothiyal K. Yuen T. W. (2004). Muscle strain and perceived exertion in patient handling with and without a transferring aid. Occupational Ergonomics, 4(3), 185–197. 10.3233/OER-2004-4304

[bibr39-00187208231211842] Kotowski S. E. Davis K. G. Marras W. S. (2022). Effectiveness of friction-reducing patient-handling devices on reducing lumbosacral spine loads in nurses: A controlled laboratory simulation study. American Journal of Safe Patient Handling & Mobility, 9(2), 77–89.

[bibr40-00187208231211842] Law M. J. Ridzwan M. I. Z. Mohd Ripin Z. Abd Hamid I. J. Law K. S. Karunagaran J. Cajee Y. (2022). REBA assessment of patient transfer work using sliding board and Motorized Patient Transfer Device. International Journal of Industrial Ergonomics, 90, 103322. 10.1016/j.ergon.2022.103322

[bibr41-00187208231211842] Lee S. J. Rempel D. (2020). Comparison of lift use, perceptions, and musculoskeletal symptoms between ceiling lifts and floor-based lifts in patient handling. Applied Ergonomics, 82, 102954. 10.1016/j.apergo.2019.102954 31546092

[bibr42-00187208231211842] Lloyd J. D. Baptiste A. (2006). Friction-reducing devices for lateral patient transfers: A biomechanical evaluation. AAOHN Journal, 54(3), 113–119. 10.1177/216507990605400304 16562622

[bibr83-00187208231211842] Marras W. S. Davis K. G. Kirking B. C. Bertsche P. K. (1999). A comprehensive analysis of low back disorder risk and spinal loading during the transferring and repositioning of patients using different techniques. Ergonomics, 42(7), 904–926. 10.1080/001401399185207. 10424181

[bibr43-00187208231211842] Marras W. S. Knapik G. G. Ferguson S. (2009). Lumbar spine forces during manoeuvring of ceiling-based and floor-based patient transfer devices. Ergonomics, 52(3), 384–397. 10.1080/00140130802376075 19296324

[bibr44-00187208231211842] Martimo K. P. Verbeek J. Karppinen J. Furlan A. D. Takala E. P. Kuijer P. P. F. Jauhiainen M. Viikari-Juntura E. (2008). Effect of training and lifting equipment for preventing back pain in lifting and handling: Systematic review. BMJ, 336(7641), 429–431. 10.1136/bmj.39463.418380.BE 18244957 PMC2249682

[bibr45-00187208231211842] Mayeda-Letourneau J. (2014). Safe patient handling and movement: A literature review. Rehabilitation Nursing: The Official Journal of the Association of Rehabilitation Nurses, 39(3), 123–129. 10.1002/rnj.133 24323744

[bibr46-00187208231211842] McGill S. M. Kavcic N. S. (2005). Transfer of the horizontal patient: The effect of a friction reducing assistive device on low back mechanics. Ergonomics, 48(8), 915–929. 10.1080/00140130412331331389 16147412

[bibr47-00187208231211842] Muona A. Vartiainen P. Karjalainen P. A. Räsänen K. (2022). Forces required in repositioning a patient in bed using regular sheet and slide film. International Journal of Industrial Ergonomics, 90, 103302. 10.1016/j.ergon.2022.103302

[bibr48-00187208231211842] Nelson A. Baptiste A. (2006). Evidence-based practices for safe patient handling and movement. OJIN: Online Journal of Issues in Nursing, 9(3), 4. 10.3912/OJIN.Vol9No03Man03 15482090

[bibr49-00187208231211842] Nelson A. Lloyd J. D. Menzel N. Gross C. (2003). Preventing nursing back injuries: Redesigning patient handling tasks. AAOHN Journal, 51(3), 126–134. 10.1177/216507990305100306 12670100

[bibr50-00187208231211842] Nelson A. L. Collins J. Knibbe H. Cookson K. De Castro A. B. Whipple K. L. (2007). Safer patient handling. Nursing Management, 38(3), 26–32. 10.1097/01.NUMA.0000262924.77680.a9 17473792

[bibr51-00187208231211842] Nevala N. Tamminen-Peter L. (2004). Ergonomics and usability of an electrically adjustable shower trolley. International Journal of Industrial Ergonomics, 34(2), 131–138. 10.1016/j.ergon.2004.03.003

[bibr52-00187208231211842] Nussbaum M. A. Torres N. (2001). Effects of training in modifying working methods during common patient-handling activities. International Journal of Industrial Ergonomics, 27(1), 33–41. 10.1016/S0169-8141(00)00037-8

[bibr53-00187208231211842] Omura Y. Hirata M. Yoshimine T. Nakatani E. Inoue T. (2022). Development and evaluation of a new assistive device for low back load reduction in caregivers: An experimental study. Scientific Reports, 12(1), 19134. 10.1038/s41598-022-21800-5 36351943 PMC9646712

[bibr54-00187208231211842] Page M. J. McKenzie J. E. Bossuyt P. M. Boutron I. Hoffmann T. C. Mulrow C. D. Shamseer L. Tetzlaff J. M. Akl E. A. Brennan S. E. Chou R. Glanville J. Grimshaw J. M. Hróbjartsson A. Lalu M. M. Li T. Loder E. W. Mayo-Wilson E. McDonald S. Moher D. (2021). The PRISMA 2020 statement: An updated guideline for reporting systematic reviews. BMJ, 372, n71. 10.1136/bmj.n71 33782057 PMC8005924

[bibr55-00187208231211842] Pluye P. Hong Q. N. (2014). Combining the power of stories and the power of numbers: Mixed methods research and mixed studies reviews. Annual Review of Public Health, 35, 29–45. 10.1146/annurev-publhealth-032013-182440 24188053

[bibr56-00187208231211842] Potvin J. (2017). An ergonomics simulation study of a clinical recliner, chair, and bed during sit-to-stand patient lifting. International Journal of Safe Patient Handling & Mobility, 7(2), 64–73.

[bibr57-00187208231211842] Riccoboni J. B. Monnet T. Eon A. Lacouture P. Gazeau J. P. Campone M. (2021). Biomechanical comparison between manual and motorless device assisted patient handling: Sitting to and from standing position. Applied Ergonomics, 90, 103284. 10.1016/j.apergo.2020.103284 33070065

[bibr58-00187208231211842] Rice M. S. Woolley S. M. Waters T. R. (2009). Comparison of required operating forces between floor-based and overhead-mounted patient lifting devices. Ergonomics, 52(1), 112–120. 10.1080/00140130802481123 19308824

[bibr59-00187208231211842] Santaguida P. L. Pierrynowski M. Goldsmith C. Fernie G. (2005). Comparison of cumulative low back loads of caregivers when transferring patients using overhead and floor mechanical lifting devices. Clinical Biomechanics, 20(9), 906–916. 10.1016/j.clinbiomech.2005.06.001 16054280

[bibr60-00187208231211842] Schibye B. Hansen A. F. Hye-Knudsen C. T. Essendrop M. Böcher M. Skotte J. (2003). Biomechanical analysis of the effect of changing patient-handling technique. Applied Ergonomics, 34(2), 115–123. 10.1016/S0003-6870(03)00003-6 12628568

[bibr61-00187208231211842] Silvia C. E. Bloswick D. S. Lillquist D. Wallace D. Perkins M. S. (2002). An ergonomic comparison between mechanical and manual patient transfer techniques. Work, 19(1), 19–34. 12454348

[bibr62-00187208231211842] Smith J. Fray M. Love J. (Eds.), (2011). The guide to the handling of people (6th ed.). BackCare, Royal College of Nursing.

[bibr63-00187208231211842] Tang R. Holland M. Milbauer M. Olson E. Skora J. Kapellusch J. M. Garg A. (2018). Biomechanical evaluations of bed-to-wheelchair transfer: Gait belt versus walking belt. Workplace Health & Safety, 66(8), 384–392. 10.1177/2165079917749862 29426267

[bibr64-00187208231211842] Teeple E. Collins J. E. Shrestha S. Dennerlein J. T. Losina E. Katz J. N. (2017). Outcomes of safe patient handling and mobilization programs: A meta-analysis. Work, 58(2), 173–184. 10.3233/WOR-172608 29036857 PMC6138450

[bibr65-00187208231211842] Theou O. Soon Z. Filek S. Brims M. Leach-MacLeod K. Binsted G. Jakobi J. (2011). Changing the sheets: A new system to reduce strain during patient repositioning. Nursing Research, 60(5), 302–308. 10.1097/NNR.0b013e318225b8aa 21873921

[bibr66-00187208231211842] Ulin S. S. Chaffin D. B. Patellos C. L. Blitz S. G. Emerick C. A. Lundy F. Misher L. (1997). A biomechanical analysis of methods used for transferring totally dependent patients. SCI Nursing: A Publication of the American Association of Spinal Cord Injury Nurses, 14(1), 19–27. 9165952

[bibr67-00187208231211842] Villeneuve J. (1998). The ceiling lift: An efficient way to prevent injuries to nursing staff. Journal of Healthcare Safety, Compliance & Infection Control, 2, 19–23.

[bibr68-00187208231211842] Vinstrup J. Jakobsen M. D. Madeleine P. Andersen L. L. (2020). Biomechanical load during patient transfer with assistive devices: Cross-sectional study. Ergonomics, 63(9), 1164–1174. 10.1080/00140139.2020.1764113 32362200

[bibr69-00187208231211842] Walls C. (2001). Do electric patient beds reduce the risk of lower back disorders in nurses? Occupational Medicine, 51(6), 380–384. 10.1093/occmed/51.6.380 11584116

[bibr86-00187208231211842] Waters T. R. (2011). Product design issues related to safe patient handling technology. In Human factors and ergonomics in consumer product design: Uses and applications (pp. 89–100). CRC Press. 10.1016/j.aorn.2010.08.025.

[bibr70-00187208231211842] Waters T. R. Dick R. Lowe B. Werren D. Parsons K. (2012). Ergonomic assessment of floor-based and overhead lifts. American Journal of Safe Patient Handling & Movement, 2(4), 119. https://pubmed.ncbi.nlm.nih.gov/26550545/26550545 PMC4631797

[bibr71-00187208231211842] Weiner C. Kalichman L. Ribak J. Alperovitch-Najenson D. (2017). Repositioning a passive patient in bed: Choosing an ergonomically advantageous assistive device. Applied Ergonomics, 60, 22–29. 10.1016/j.apergo.2016.10.007 28166880

[bibr72-00187208231211842] Wiggermann N. (2016). Biomechanical evaluation of a bed feature to assist in turning and laterally repositioning patients. Human Factors, 58(5), 748–757. 10.1177/0018720815612625 26715690

[bibr73-00187208231211842] Wiggermann N. Zhou J. McGann N. (2021). Effect of repositioning aids and patient weight on biomechanical stresses when repositioning patients in bed. Human Factors, 63(4), 565–577. 10.1177/0018720819895850 31999485 PMC8114440

[bibr88-00187208231211842] Willey M. S. (2001). The effects of back belts and load on selected lifting kinematics during a simulated patient transfer. Work, 17(1), 31–38. 12441620

[bibr74-00187208231211842] Wilson T. P. Davis K. G. (2016). Health care ergonomics: Contributions of Thomas Waters. Human Factors, 58(5), 726–747. 10.1177/0018720816648553 27268995

[bibr75-00187208231211842] Zakerian S. A. Afzalinejhad M. Mahmodi M. Sheibani N. (2021). Determining the efficiency of ergonomic belt during patient handling and its effect on reducing musculoskeletal disorders in nurses. SAGE Open Nursing, 7, 23779608211057939. 10.1177/23779608211057939 34888415 PMC8649436

[bibr87-00187208231211842] Zhou J. Wiggermann N. (2021). The effects of hospital bed features on physical stresses on caregivers when repositioning patients in bed. Applied Ergonomics, 90, 103259. 10.1016/j.apergo.2020.103259. 32977144

[bibr76-00187208231211842] Zhuang Z. Stobbe T. J. Hsiao H. Collins J. W. Hobbs G. R. (1999). Biomechanical evaluation of assistive devices for transferring residents. Applied Ergonomics, 30(4), 285–294. 10.1016/S0003-6870(98)00035-0 10416841

